# Supplementary material for: A high-resolution mRNA expression time course of embryonic development in zebrafish
Source: eLife. 2017 Nov 16;6:e30860. doi: 10.7554/eLife.30860 (PMC5690287; doi:10.7554/eLife.30860)
Supplement: Supplementary file 6. [file elife-30860-supp6.zip › biolayout-clusters-files/Cluster160-genes.html]

Cluster160


# Cluster160: Genes

| Ensembl ID | Gene Name | Chr | Start | End | Biotype |
| --- | --- | --- | --- | --- | --- |
| ENSDARG00000097530 | ENSDARG00000097530 | 17 | 32655499 | 32668592 | protein\_coding |
| ENSDARG00000058488 | adora2ab | 21 | 13196723 | 13217280 | protein\_coding |
| ENSDARG00000030449 | crabp2b | 19 | 9054117 | 9061113 | protein\_coding |
| ENSDARG00000104566 | foxi1 | 12 | 43916243 | 43918010 | protein\_coding |
| ENSDARG00000054509 | hesx1 | 11 | 42344031 | 42345373 | protein\_coding |
| ENSDARG00000013853 | lmo4a | 5 | 32324516 | 32346102 | protein\_coding |
| ENSDARG00000009471 | nid2b | 20 | 9656474 | 9687637 | protein\_coding |
| ENSDARG00000035521 | sfrp1a | 5 | 26888822 | 26938592 | protein\_coding |
| ENSDARG00000101717 | sox17 | 7 | 58469813 | 58473735 | protein\_coding |
